# New insight into avian malaria vectors in New Zealand

**DOI:** 10.1186/s13071-024-06196-7

**Published:** 2024-03-22

**Authors:** E. R. Schoener, D. M. Tompkins, L. Howe, I. C. Castro

**Affiliations:** 1 School of Natural Sciences (SNS), Ecology, Massey University, Palmerston North, New Zealand; 2 Present Address: Laboklin-Labor Für Klinische Diagnostik GMBH& Co. KG, Abteilung Molekularbiologie, Bad Kissingen, Germany; 3 Predator Free 2050 Limited, Auckland, New Zealand; 4 School of Veterinary Science, Tāwharau Ora, Massey University, Palmerston North, New Zealand

**Keywords:** *Aedes*, *Culex*, Mosquito, *Plasmodium*

## Abstract

**Background:**

Mosquitoes (Culicidae) are vectors for most malaria parasites of the *Plasmodium* species and are required for *Plasmodium* spp. to complete their life cycle. Despite having 16 species of mosquitoes and the detection of many *Plasmodium* species in birds, little is known about the role of different mosquito species in the avian malaria life cycle in New Zealand.

**Methods:**

In this study, we used nested polymerase chain reaction (PCR) and real-time PCR to determine *Plasmodium* spp. prevalence and diversity of mitochondrial *cytochrome b* gene sequences in wild-caught mosquitoes sampled across ten sites on the North Island of New Zealand during 2012–2014. The mosquitoes were pooled by species and location collected, and the thorax and abdomens were examined separately for *Plasmodium* spp. DNA. Akaike information criterion (AIC) modeling was used to test whether location, year of sampling, and mosquito species were significant predictors of minimum infection rates (MIR).

**Results:**

We collected 788 unengorged mosquitoes of six species, both native and introduced. The most frequently caught mosquito species were the introduced *Aedes notoscriptus* and the native *Culex pervigilans*. *Plasmodium* sp DNA was detected in 37% of matched thorax and abdomen pools. When considered separately, 33% of abdomen and 23% of thorax pools tested positive by nested PCR. The MIR of the positive thorax pools from introduced mosquito species was 1.79% for *Ae. notoscriptus* and 0% for *Cx. quinquefasciatus*, while the MIR for the positive thorax pools of native mosquito species was 4.9% for *Cx. pervigilans* and 0% for *Opifex fuscus*. For the overall MIR, site and mosquito species were significant predictors of *Plasmodium* overall MIR. *Aedes notoscriptus* and *Cx. pervigilans* were positive for malaria DNA in the thorax samples, indicating that they may play a role as avian malaria vectors. Four different *Plasmodium* lineages (SYAT05, LINN1, GRW6, and a new lineage of *P (Haemamoeba)* sp. AENOT11) were identified in the pooled samples.

**Conclusions:**

This is the first detection of avian *Plasmodium* DNA extracted from thoraxes of native *Culex* and introduced *Aedes* mosquito species in New Zealand and therefore the first study providing an indication of potential vectors in this country.

**Graphical Abstract:**

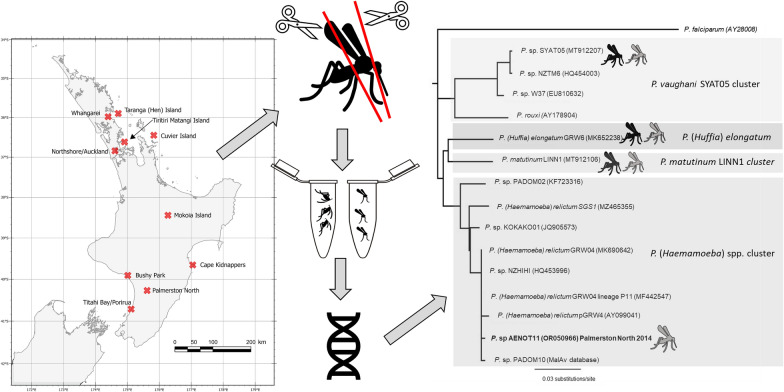

## Background

Avian malaria parasites of the genus *Plasmodium* are common in birds worldwide. Competent insect vectors are compulsory for malaria parasites to complete their life cycle. Following several cycles of schizogony (asexual reproduction) in the vertebrate host, the *Plasmodium* sexual stages, namely gametocytes or gamonts, start to develop in mature erythrocytes. These sexual stages are acquired by the vector when they feed on bird blood [1] and develop into macro- and micro-gametes in the insect’s gut within the abdomen of the mosquito. After sexual reproduction and one period of asexual reproduction (sporogony), sporozoites travel through the hemocele of the vector and penetrate the salivary glands in the thorax of the mosquito. From there, they are transmitted by the insects to the avian host during blood feeding [2–4]. Different *Plasmodium* parasites can be transmitted by a range of mosquito species. For example, in Hawaii, a culicine mosquito, *Culex quinquefasciatus,* is the main, but not sole, vector for *Plasmodium relictum* lineage GRW4, with two other mosquitoes, *Aedes albopictus* and *Wyeomyia mitchellii,* also implicated, although transmissibility to confirm vector competence was never tested [5].

Avian malaria parasites of 17 different lineages have been found in 37 different bird species in New Zealand [6, 7]. The lineages most frequently detected infecting native and introduced birds are *Plasmodium* (*Huffia*) *elongatum* GRW6 with the widest host range, *Plasmodium matutinum* LINN1, and *Plasmodium vaughani* SYAT05. Other lineages of *Plasmodium* detected in endemic species include *P. relictum* lineages GRW4 and SGS1 [6]. The majority of the 17 lineages are thought to have been brought into New Zealand with the many species of introduced passerines from Europe [8, 9]. However, three native *Plasmodium* lineages have also been reported: *P.* sp. KOKAKO01/PADOM02 [10], *P.* (*Haemamoeba*) lineage NZHIHI [6], and *P.* sp. BELL01 [9, 11].

Mortality due to *Plasmodium* species infection in New Zealand has been recorded mostly in captive situations; for example, in New Zealand dotterel (*Charadrius obscurus*) chicks in 1996 [12], yellowhead/mohua (*Mohua ochrocephala*) in 2004 [13], brown kiwi (*Apteryx mantelli*) in 2010/2011 [14], and little penguins (*Eudyptula minor*) [7]. In addition, there is one confirmed instance of mortality in a reintroduced, wild population of South Island saddlebacks (*Philesturnus carunculatus carunculatus*) [15]. To date, confirmed mortalities due to *Plasmodium* spp. in native New Zealand birds have been from infections with the introduced *Plasmodium* lineages GRW6, LINN1 and a native *P.* (Haemamoeba) lineage NZHIHI [6].

New Zealand possesses 12 species of native mosquitoes (genus: *Culex*, *Culiseta*, *Coquillettidia*, *Opifex*, *Aedes*, *Maorigoeldia*) and four introduced species (genus: *Culex*, *Aedes*) [16, 17], but there is limited information as to which mosquito species are vectors of avian malaria and whether there is any vector-*Plasmodium* species specificity [8, 18, 19]. A distribution study by Tompkins and Gleeson [8] showed a strong negative correlation between *Plasmodium* spp. prevalence in birds and longitude, closely matching the known distribution of the invasive *Cx. quinquefasciatus*, which is thought to have been introduced in the 1880s. However, Gudex-Cross et al. [20] demonstrated that invasive mosquito species were almost exclusively present on the forest edge of a New Zealand regional park, despite a similar *Plasmodium* spp. infectious prevalence in birds within the forest edge and the interior habitats. These findings, together with the existence of native *Plasmodium* lineages, indicate a role of native mosquitoes as vectors for *Plasmodium* species. Only one study to date, by Massey et al. [21], found avian *Plasmodium* DNA in the abdomen of one engorged female of the native mosquito *Cx. pervigilans.* However, vector status could not be determined.

The aim of this study was thus to expand on the limited knowledge of role of mosquitoes in the transmission of avian malaria in New Zealand by assessing the presence of different *Plasmodium* species separately in their thorax and abdomen. *Plasmodium* presence in the thorax, where the salivary glands are located, is a stronger indicator of vector competence [43]. We also examined whether location, year of sampling, and mosquito species were significant predictors of infection rates (MIR).

## Methods

### Sampling locations

**Fig. 1:**
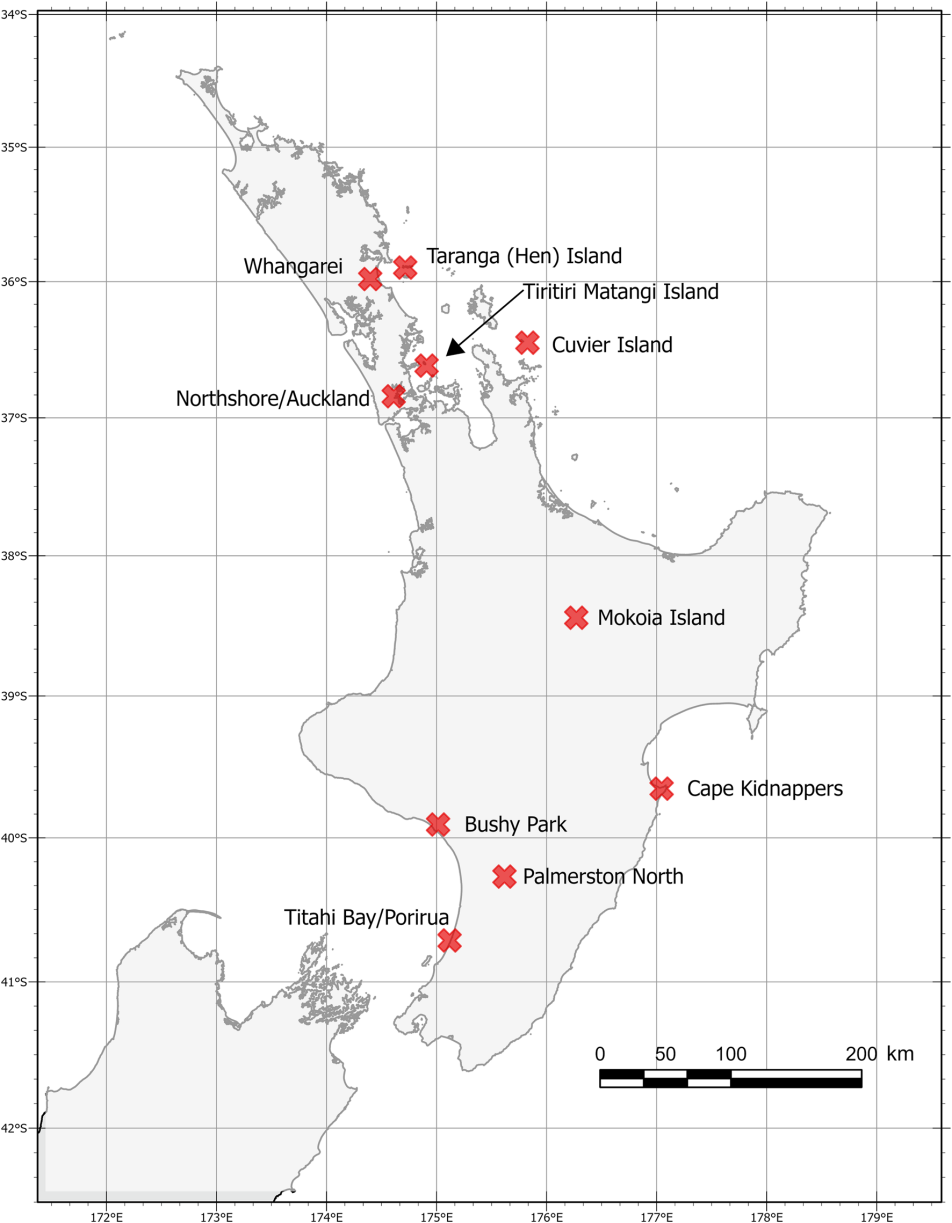
Location of sites around the North Island of New Zealand where mosquito species were captured for the detection of *Plasmodium* spp. DNA

During the period 2012–2014, a total of 13 sampling trips were carried out to catch mosquitoes at the following sites in the North Island of New Zealand from North to South: Whangarei, Hen Island, Cuvier Island, Tiritiri Matangi Island, Auckland/Northshore, Mokoia Island, Cape Kidnappers, Bushy Park, Palmerston North, and Titahi Bay/Porirua (Fig. 1). These sites comprised conservation islands and mainland sites where endangered native species have been translocated, as well as non-conservation public sites in the nearby mainland. Traps were set up along trails and around campsites in sites ranging from uninhabited forested reserves and campgrounds to an urban wetland (Table 1).

**Table 1:** Locations, with a brief site description, and date of sampling of mosquitoes within the North Island of New Zealand

Location	Site use	Size (ha)	Latitude	Sampling date	Season	Habitat and sampling site
Mokoia Island	Bird sanctuary: limited access for tour parties	135	38°05′S	March 2012 Feb 2013 February 2014	Autumn Late summer Late summer	Island on an inland lake. Natural regenerating forest for the last 57 years; 25% of land area covered in grasslands. Sampling occurred along the walking paths in the forest, around the campsite, and public toilets
Hen Island	Nature reserve: access restricted	470	35°91’S	November 2012	Spring	Offshore Island. Coastal broadleaf native forest; regenerating in areas modified by previous Māori occupation. Sampling occurred in the forest around the campsite at Dragonmouth Cove
Tiritiri Matangi Island	Open sanctuary; visitors allowed, heavy biosecurity	220	36°60’S	February 2013	Late summer	Offshore island; 60% forest in early stages of reforestation as part of restoration programme, 40% grassland. Sampling occurred in the forest surrounding the human settlement
Cape Kidnappers	Fenced native bird sanctuary: visitors allowed, heavy biosecurity	2500	39°64´ S	February 2013	Late summer	Peninsula. Scrubland, commercial pine, and naturally regenerating native coastal forest. Sampling was conducted at the area designated as the release site for the translocated North Island saddleback
Whangarei Mair Park	Public urban gardens	UB*	35°43'S	February 2013	Late summer	Mainland. Public urban gardens. Mixture native and garden plants
Auckland Northshore	Public urban gardens	UB	36°84´S	February 2013	Late summer	Mainland. Mixture native and garden plants
Bushy Park	Fenced native bird sanctuary; visitors allowed, heavy biosecurity	98	39°57`S	February 2013 February 2014	Late summer Late summer	Mainland. Mature native forest remnant. Sampling occurred in the forest along the walking tracks including in a swampy pond area
Titahi Bay	Public land	UB	41°09´S	March 2013	Autumn	Coast. Sampling occurred around tidal rock pools
Cuvier Island	Restricted Nature Reserve: access allowed with permission from the local DOC office	170	36°26’S	April 2013	Autumn	Offshore island. Naturally regenerating coastal forest. Sampling occurred around the historic human settlement, which has a small stream and a limited number of artificial freshwater containers
Palmerston North	Privately own land	UB	40°21′S	January 2014	Summer	Mainland. Sampling occurred at an urban site with bushy native scrub vegetation and a small swampy area

*UB indicates an area unbounded and lacking a natural or man-made boundary

### Trapping of mosquitoes

In most cases, sampling was scheduled for summer and/or autumn because mosquitoes caught in summer and autumn are more likely to be infected than those caught in spring [22, 23]. During the summer 2012/2013, dry weather with drought conditions in large parts of the North Island had a negative impact on the number of mosquitoes caught. To increase sample size, sampling was therefore repeated in January/February 2014 (summer) at Mokoia Island, Bushy Park, Titahi Bay, and Palmerston North because these sites were easy to access.

Mosquitoes were trapped using Centres for Disease Control and Prevention (CDC) traps baited with CO_2_ (dry ice) and UV-diode light such as the BioQuip dry ice traps [24, 25]. The traps were placed approximately 1.5 m high in trees or shrubs. Since most of the mosquito species present in the North Island appear to be crepuscular and/or nocturnal feeders [26], the traps were set approximately 2 hours before sunset (19:00 h) and emptied approximately 2 hours after sunrise (07:00 h) for a total of 12 trapping hours per sampling session. In Titahi Bay, mosquitoes were collected on one afternoon (March 2013) with insect aspirators from around the rock pools. Any female mosquitoes collected in the traps were pooled by date and site location and placed into 1.5 ml microtubes. The mosquitoes were killed by freezing at −20 °C and stored in 80% ethanol until processed for identification and DNA extraction.

### DNA extraction

Mosquitoes were identified under a stereomicroscope using the Snell [17] identification keys. Mosquitoes were dissected using a micro-scalpel in 0.9% saline into head, abdomen, and thorax and then separated into species by location and date. Abdomens were dissected first to limit potential contamination by accidental damage to the salivary glands. Next, the heads and thorax were separated. Heads were examined to ensure salivary glands remained in the thorax. The heads were then discarded to avoid introducing PCR inhibitors [27]. Instruments were cleaned with 75% ethanol between individuals and sterilized with the flame of a Bunsen burner to prevent tissue contamination between samples. Not all mosquitoes could be identified to species and genus level as the storage in ethanol caused distortion in some distinguishing features. These unidentified mosquitoes were pooled separately by site and date of collection. Dissected tissues were then pooled by species, ranging from 1 to 20 mosquitoes, depending on the number of each species at each site and sampling period. The insect parts in the species pools were then homogenized with the aid of plastic micro-pestle. The DNA from the abdomen and thorax was extracted separately using the DNeasy Blood and Tissue kit (Qiagen, Düsseldorf, Germany) according to the manufacturer’s instructions with the addition of 30 µl of 100 mg⁄mL dithiothreitol added to the 200 μl of digestion buffer to help dissolve the hard exoskeleton [28] and total DNA was eluted in the final step with 200 µl of elution buffer provided in the kit.

### PCR and sequencing

DNA from mosquito abdomen or thorax samples were subjected to an internationally accepted nested PCR for detecting avian Haemosporida parasites using the primer sets HeamNFI/HeamNR2 and HeamF/HeamR2 [29] as previously described [10]. After PCR amplification, all the amplicons were run on a 1% (w/v) ultra-pure agarose gel (Invitrogen, California, USA) containing ethidium bromide and visualized under ultraviolet light on a transilluminator. All positive PCR amplicon samples were purified (PureLink PCR purification kit, Invitrogen, California, USA) and subjected to automatic dye-terminator cycle sequencing with BigDye™ Terminator Version 3.1 Ready Reaction Cycle Sequencing kit and the ABI3730 Genetic Analyzer (Applied Biosystems Inc, California, USA) to confirm genomic sequence. The electropherograms resulting from sequencing were also checked for double nucleotide peaks to infer possible cases of mixed infections of two or more different parasite lineages. The *Plasmodium* isolate sequences obtained were compared with the MalAvi database [30] and by NCBI Blast to those other published sequences available from GenBank. The resulting sequences were submitted to the GenBank database (OR050956-OR050966).

The 11 mitochondrial *cytochrome b* sequences from our survey and 18 reference sequences for the MalAvi and GenBank databases, including representatives of the *P.* (*Huffia*) *elongatum, P.* (*Haemamoeba*) *relictum*, and *P*. (*Novyella*) spp. lineages, as proposed by Valkiunas et al. [31, 32], were trimmed to the same length (450 bases) using Geneious™ (Biomatters, Auckland, New Zealand) and aligned using Clustal W [33]. A phylogenetic tree and sequence divergence was calculated as described by Howe et al. [10].

### Presence of several different lineages in one sample

Samples that were positive on the standard nested PCR were reexamined using a real-time PCR (qPCR) protocol using the primers HRMF [34] and HaemR2 [29] amplifying a 127 bp product of the *cytochrome b* gene as described by Schoener et al. [35]. This qPCR method can discern between the most common *Plasmodium* lineages in New Zealand [*P. elongatum* GRW6 (GenBank MK652238), *P. matutinum* LINN1 (GenBank MT912106), and *P. vaughani* SYAT05 (GenBank MT912207) as well as *P. relictum* GRW4 (GenBank AY099041)] to give an indication of possible presence of several different lineages within the same sample.

### Statistical analysis

The minimum infection rate (MIR) of each mosquito species was calculated as described by White et al. [36] to evaluate the infection rate of the collected mosquitoes. If a mosquito pool was positive for *Plasmodium* DNA on PCR, it was assumed that the pool contained at least one positive individual. Minimum infection rate was calculated for positive abdomen + thorax and thorax only pools. Therefore, MIR (percentage) was calculated as follows:

$${\text{MIR}}\,(\% ) = \frac{{n_{{({\text{PCR}}\,{\text{positive}}\,{\text{pools}})}} }}{{n_{{({\text{total}}\,{\text{analysed}}\,{\text{mosquitoes}})}} }} \times 100$$

The small sample size and the unbalanced dataset, due to the nature of our sampling, limited the number of potentially meaningful statistical models that could be used. For this reason, we used model simplification [37] to compare, using the Akaike information criterion (AIC), a set of generalized linear models (GLM). The full model examined the predictive value of mosquito species (two most common species: *Aedes notoscriptus* and *Culex pervigilans*), site (Auckland, Bushy Park, Cape Kidnappers, Cuvier, Hen, Mokoia, Palmerston North, Titahi Bay, and Whangarei), and year of sampling (2012, 2013, and 2014) on overall MIR and thorax MIR. The number of mosquito pools was used as a covariate in the models. We accepted the model with the lowest AIC as the one that best predicted the MIR data. When this model and the next model were within two AIC points, this model was also highlighted (Greenwood 2023-8.13.1—https://libretexts.org/—book downloaded 10-01-2024).

Owing to the low sample sizes of certain mosquito species, we did not test for differences in the parasite prevalence among mosquito species. Tests were carried out in IBM SPSS statistics (Version 28.0.0.0, Armonk, New York).

## Results

### Mosquito Fauna

We collected a total of 788 mosquitoes of six species at nine sites (Table 2). All mosquitoes were unengorged. No mosquitoes were caught on Tiritiri Matangi. In Titahi Bay, only the endemic *Opifex fuscus* was collected. The most common mosquito species captured was the introduced *Ae. notoscriptus* with 70.5% (556/788) of the total individuals caught (Table 2). This species was found at all sites where CO_2_-baited traps were used but it was most common in urban areas where it dominated the collected community, with 100% of the caught mosquitoes in Whangarei (9/9) and Auckland (21/21) as well as 94% (442/472) in Palmerston North being of this species (Table 2). The native *Cx. pervigilans* was the second most common mosquito found, with 12.9% (102/788) of the individuals caught, but was only present at 4/10 sites. This mosquito was more prominent in nature reserves and predominated in March 2012 on Mokoia Island (49.15%, 29/59) and in February 2013 in Bushy Park (61.61%, 61/99). Other species identified included the native *Ae.* (*Ochlerotatus*) *antipodeus* (34 individuals) and *Cx. astelidae* (1 individual), the introduced *Cx. quinquefasciatus* (11 individuals)*,* two individuals which could only be identified at the genus level (*Culex* spp.), and 39 individuals that could not be identified at the genus level due to loss of key features while in ethanol storage (Table 2).

**Table 2:** Species and numbers of mosquitoes sampled during this study arranged by date of sampling. Dashes indicate that a species was not caught at the location

Location	Sampling date	Mosquito species								Number of individuals
		Introduced		Native				Unidentified		
		*Aedes notoscriptus*	*Culex quinquefasciatus*	*Aedes antipodeus*	*Culex pervigilans*	*Opifex fuscus*	*Culex astelidae*	*Culex* spp	Unknown spp	
Mokoia Is	March 2012	20	1	9	29	0	0	0	0	59
	February 2013	0	0	0	0	0	0	0	0	0
	February 2014	20	3	4	0	0	0	2	0	29
Hen Is	November 2012	3	0	8	0	0	1	0	0	12
Cape Kidnappers	February 2013	1	0	0	5	0	0	0	0	6
Whangarei Mair Park	February 2013	9	0	0	0	0	0	0	0	9
Tiritiri Matangi Is	February 2013	0	0	0	0	0	0	0	0	0
Auckland Northshore	February 2013	21	0	0	0	0	0	0	0	21
Bushy Park	February 2013	12	0	4	61	0	0	0	22	99
	February 2014	8	0	9	1	0	0	0	0	18
Titahi Bay	March 2013	0	0	0	0	24	0	0	0	24
Cuvier Island	April 2013	20	0	0	0	19	0	0	0	39
Palmerston North	January 2014	442	7	0	6	0	0	0	17	472
Individuals collected, total		556	11	34	102	43	1	2	39	788

There were differences in mosquito species composition at the two sites where several sampling trips were conducted in consecutive years. On Mokoia Island, in March 2012, *Ae. notoscriptus* was most prevalent, while in February 2013 no mosquitoes were caught, and in February 2014 *Cx. pervigilans* was the most common species. In Bushy Park, *Ae. notoscriptus* was predominant in February 2013, while 1 year later in February 2014, the native *Cx. pervigilans* was more common (Table 2).

### Potential avian malaria vectors

When the thorax and abdomen pools were matched and results combined, there was a minimal *Plasmodium* sp DNA prevalence of 37% (28/75), where a matched pool was considered positive if either the thorax or abdomen pool was PCR positive, but not counted twice if both were positive. A total of 42 (28%) of 150 (75 of each abdomen and thorax) were positive for *Plasmodium* spp. DNA (Table 3). Of these, 25/75 (33%) abdomen and 17/75 (23%) thorax pools tested positive. Avian malaria parasite DNA was found in four different mosquito species. However, of these four species, only two, the native *Cx. pervigilans* and introduced *Ae. notoscriptus,* had both thorax and abdomen pools that tested positive (Table 3).

**Table 3:** *Plasmodium* spp. DNA identified in collected mosquito pools (a, abdomen; t, thorax); the sites are arranged by time of sampling

Location	Sampling date	*Number of positive pools per mosquito species*								Total number of matched positive pools	
		Introduced		Native				Unidentified			
		*Aedes notoscriptus*	*Culex quinquefasciatus*	*Culex pervigilans*	*Opifex fuscus*	*Aedes antipodeus*	*Culex astelidae*	*Culex spp*	Unknown spp.		
Mokoia Is	March 2012	0/1(a) 0/1(t)	0/1(a) 0/1(t)	**2/2(a) 2/2(t)**	–	0/1(a) 0/1(t)	–	–	–		**2/5 (40%)**
	Feb-14	0/2(a) 0/2(t)	0/1(a) 0/1(t)	–	–	0/1(a) 0/1(t)	–	0/1(a) **1/1(t)**	–		**1/5 (20%)**
Hen Is	November 2012	**1/1(a)** 0/1(t)	–	–	–	0/1(a) 0/1(t)	0/1(a) 0/1(t)	–	–		**1/3 (33%)**
Cape Kidnappers	February 2013	0/1(a) **1/1(t)**	–	**1/1(a)** 0/1(t)	–	–	–	–	–		**2/2 (100%)**
Whangarei Mair Park	February 2013	0/1(a) 0/1(t)	–	–	–	–	–	–	–		**0/1 (0%)**
Auckland Northshore	February 2013	0/1(a) 0/1(t)	–	–	–	–	–	–	–		**0/1 (0%)**
Bushy Park	February 2013	**1/1(a) 1/1(t)**	–	**3/3(a) 1/3(t)**	–	0/1(a) 0/1(t)	–	–	**2/2(a)** 0/2(t)		**6/7 (86%)**
	February 2014	0/1(a) 0/1(t)	–	0/2(a) **1/2(t)**	–	0/1(a) 0/1(t)	–	–	–		**1/4 (25%)**
Titahi Bay	March 2013	–	–	–	**1/2(a)** 0/2(t)	–	–	–	–		**1/2 (50%)**
Cuvier Island	April 2013	0/3(a) 0/3(t)	–	–	0/2(a) 0/2(t)	–	–	–	–		**0/5 (0%)**
Palmerston North	January 2014	**10/35(a) 8/35(t)**	**1/1(a)** 0/1(t)	**2/2(a) 1/2(t)**	–	–	–	–	**1/2(a) 1/2(t)**		**14/40 (35%)**
**Total matched pools** **Total pools (a + t)** **Individual pools**		**13/47 (28%)** **22/94 (23%)** **12/47(a) 10/47(t)**	**1/3 (33%)** **1/6 (17%)** **1/3(a)** 0/3(t)	**9/10 (90%)** **13/20 (65%)** **8/10(a) 5/10(t)**	**1/4 (25%)** **1/8 (13%)** **1/4(a)** 0/4(t)	0/5 (0%) 0/10 (0%) 0/5(a) 0/5(t)	0/1 (0%) 0/2 (0%) 0/1(a) 0/1(t)	1/1 (100%) 1/2 (50%) 0/1(a) **1/1(t)**	**3/4 (75%)** **4/8 (50%)** **3/4(a) 1/4(t)**		**28/75 (37%)** **42/150 (28%)** **25/75(a) 17/75(t)**

The *Plasmodium* MIR of the thorax positive pools was highest for *Cx. pervigilans* (4.9%), followed by *Ae. notoscriptus* (1.79%). Although abdominal pools for both *Cx. quinquefasciatus* and *Opifex fuscus* were positive, the thorax pools were negative, resulting in an 0% thorax MIR (Table 4). When the simplified AIC models were considered, the two models including only site and mosquito species + site were significant predictors of *Plasmodium* overall MIR for the two most captured mosquito species sampled (Table 5). For the thorax MIR, the model with only mosquito species and the model with only year as fixed factors were the best for predicting its values (Table 5).

**Table 4:** Minimum infection rate (MIR) for collected mosquitoes

Mosquito species	Number of mosquitoes	Positive matched pools (a + t)	MIR overall (%)	Positive thorax pools	MIR thorax (%)
Introduced					
*Aedes notoscriptus*	556	22	3.96	10	1.79
*Culex quinquefasciatus*	11	1	9.09	0	0
Native					
*Opifex fuscus*	43	1	2.32	0	0
*Culex pervigilans*	102	13	12.75	5	4.90

**Table 5:** Model comparison and statistical details for factors used as possible predictors of overall (mosquito) or thorax mosquito infection rate (MIR) in generalized linear models

Model	Source	Wald chi-squared	*df*	*P*-value		AIC
Overall MIR						
Mosquito species, site, year	(Intercept)	5.812	1	**0.016**		139.2
	Mosquito species	3.608	1	0.057		
	Site	43.411	7	** < 0.001**		
	year	2.909	2	0.234		
	Pool number	13.768	1	** < .001**		
Mosquito species, site	(Intercept)	16.300	1	** < 0.001**		137.85
	Mosquito species	1.396	1	0.237		
	Site	33.920	7	** < 0.001**		
	Pool number	9.622	1	**0.002**		
Mosquito species, year	(Intercept)	5.613	1	**0.018**		144.96
	Mosquito species	0.390	1	0.532		
	Year	0.114	2	0.945		
	Pool number	0.341	1	0.56		
Site, year	(Intercept)	9.261	1	**0.002**		140.41
	Site	32.919	7	** < 0.001**		
	Year	0.785	2	0.676		
	Pool number	8.176	1	**0.004**		
Mosquito species	(Intercept)	6.532	1	**0.011**		141.07
	Mosquito species	0.374	1	0.541		
	Pool number	0.302	1	0.583		
Year	(Intercept)	5.094	1	**0.024**		143.35
	Year	0.098	2	0.952		
	Pool number	0.420	1	0.517		
Site	(Intercept)	21.116	1	** < 0.001**		137.18
	Site	30.740	7	** < 0.001**		
	Pool number	7.481	1	**0.006**		
Thorax MIR						
Mosquito species, site, year	(Intercept)	0.023	1	0.879		141.41
	Mosquito species	5.709	1	**0.017**		
	Site	19.111	7	**0.008**		
	Year	1.800	2	0.407		
	No. pools	2.629	1	0.105		
Mosquito species, site	(Intercept)	0.760	1	0.383		141.05
	Mosquito species	3.572	1	0.059		
	Site	1.608	1	0.205		
	No. pools	16.870	7	**0.018**		
Mosquito species, year	(Intercept)	0.785	1	0.376		141.41
	Mosquito species	0.118	1	0.732		
	Year	0.731	2	0.694		
	No. pools	0.065	1	0.799		
Site, year	(Intercept)	0.971	1	0.324		146.14
	Site	9.717	7	0.205		
	Year	0.087	2	0.958		
	No. pools	0.316	1	0.574		
Mosquito species	(Intercept)	1.522	1	0.217		138.12
	Mosquito species	0.133	1	0.715		
	No. pools	0.180	1	0.672		
Year	(Intercept)	1.081	1	0.299		139.52
	Year	0.748	2	0.688		
	No. pools	0.047	1	0.828		
Site	(Intercept)	2.285	1	0.131		142.23
	Site	10.830	7	0.146		
	No. pools	0.295	1	0.587		

### *Plasmodium* lineages

A total of 42 mosquito species pools were positive for the presence of *Plasmodium* spp. However, only 30 of these could be sequenced with conclusive results (Tables 6 and 7). Of these, analysis of the electropherograms from direct sequencing or the qPCR results revealed that 16 (53.33%) pools carried more than one *Plasmodium* lineage (Tables 6 and 7). Of note, only four of these mixed pools were identified by both the nested PCR and qPCR.

**Table 6:** Plasmodium lineages found in collected mosquito pools (a, abdomen; t, thorax)

Mosquito species	Pools positive for *Plasmodium* spp. DNA	Number of pools successfully sequenced	Plasmodium lineages				Number of pools with multiple lineages identified
			LINN1	GRW6	SYAT05	*Relictum* spp.	
Introduced							
*Aedes notoscriptus*	22	17	9 (6a;3t)	1(t)	10 (4a;6t)	1(a)	8
*Culex quinquefasciatus*	1	1	1(a)	1(a)			1
Native					–	–	
*Culex pervigilans*	13	9	7 (5a;2t)	6 (4a;2t)	3(2a;1t)	–	6
*Opifex fuscus*	1	0	–	–	–	–	0
*Aedes antipodeus*	0	0	–	–	–	–	0
*Culex astelidae*	0	0	–	–	–	–	0
Unidentified							
*Culex* species	1	1	–	–	1(t)	–	0
Unknown species	4	2	1(a)	–	1(a)	–	1
Total	42	30	18 (60%) (14a; 6t)	8 (26.7%) (5a; 4t)	15 (50%) (8a; 8t)	1 (3.3%) (1a)	16

**Table 7:** *Plasmodium* species lineages found at sites (a, abdomen; t, thorax)

Location	Pools positive for *Plasmodium* spp. DNA	Number of pools successfully sequenced	*Plasmodium* spp. lineages				Number of pools with multiple lineages identified
			LINN1	GRW6	SYAT05	*Relictum* spp.	
Hen Island	1	1	–	–	1(1a)	–	0
Cape Kidnappers	2	2	–	1 (1t)	1 (1a)	–	1
Mokoia Island	5	3	2(1a;1t)	2(1a;1t)	4(2a;2t)	–	2
Bushy Park	9	7	5(4a;1t)	2(2a)	1(1a)	–	3
Palmerston North	24	17	11(8a;3t)	3(2a;1t)	8(2a;6t)	1(a)	10
Titahi Bay	1	NS	–	–	–	–	0
Total	42	30	18 (60%) (13a; 5t)	8 (26.7%) (5a; 3t)	15 (50%) (7a; 8t)	1 (3.3%) (1a)	16

Four different introduced *Plasmodium* lineages were identified, namely *P. matutinum* LINN1 (18/30, 99.6–100% sequence homology to GenBank MT912106), *P. vaughani* SYAT05 (15/30, 99.8–100% sequence homology to GenBank MT912207), *P.* (Huffia) *elongatum* GRW6 (8/30, 100% sequence homology to GenBank MK652238), and one case of a previously undescribed *P. (Haemamoeba)* isolate (lineage AENOT11) (Table 6). Both *Ae. notoscriptus* and *Cx. pervigilans* carried the *Plasmodium* lineages LINN1, SYAT05, and GRW6 in both the abdomen and thorax. A single pool from the abdomen of *Ae. notoscriptus* was found to carry the novel lineage AENOT11 (Table 6).

Three of the *Plasmodium* lineages LINN1, SYAT05, and GRW6 had widespread prevalence and were found on three of the six *Plasmodium *spp. positive sites around the North Island (Table 7). For the three additional positive sites, Cape Kidnappers had only *Plasmodium* lineages SYAT05 and GRW6 detected, Hen Island had only *P. vaughani* SYAT05 detected, and one positive mosquito pool from Titahi Bay could not be genotyped. The novel *P.* lineage AENOT11 was only detected in Palmerston North (Table 7), which was the largest sample of mosquitoes obtained. There was no significant difference in lineage diversity between sites (*χ*^2^ = 7.27, *df* = 10, *P* > 0.05).

**Fig. 2:**
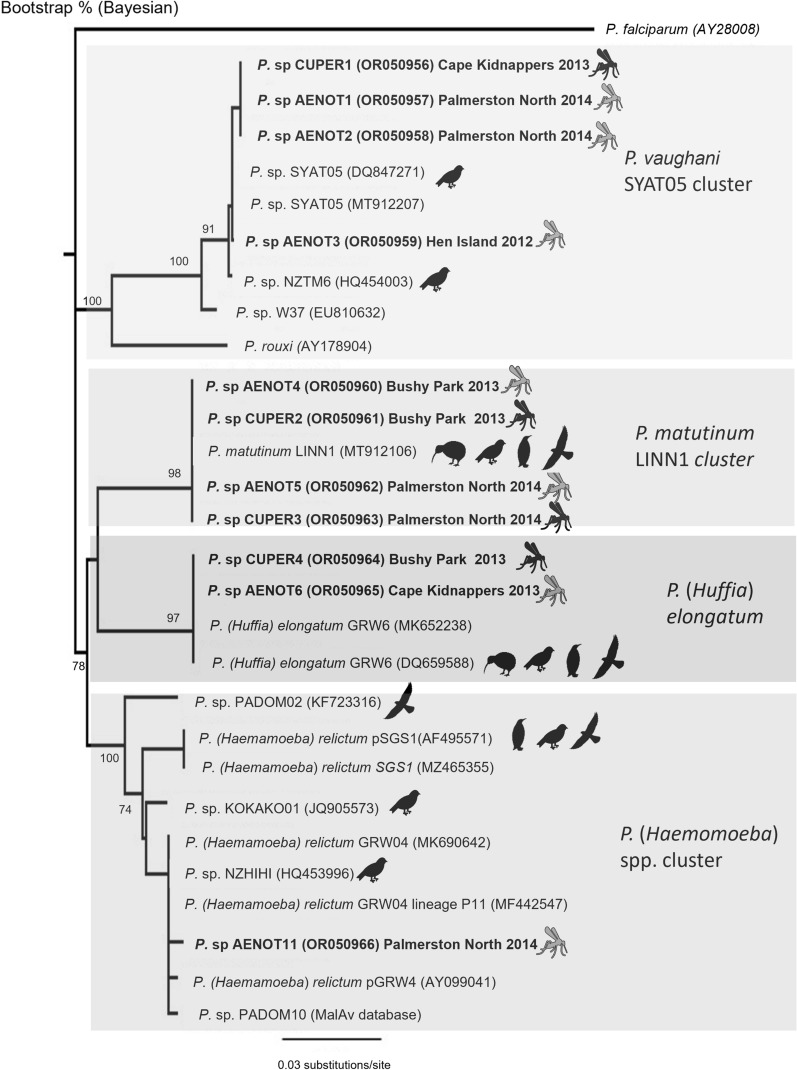
Bayesian phylogenetic analysis and comparison of 11 *Plasmodium* spp. isolates (bold) from *Aedes notoscriptus (**)*, *Culex pervigilans* (), and previously published *Plasmodium* spp. sequences present in the GenBank and/or MalAvi database. Avian icons represent previous reports of *Plasmodium* lineages identified in New Zealand avifauna, including kiwi (), raptor species (), penguins (), and a variety of Passeriformes (). *Plasmodium falciparum* used as an outgroup. Numbers above or below branch nodes indicate bootstrap support based on 1000 replicates. Names of the lineages (when available) and GenBank accession numbers of the sequences are given after the species names of the parasites. The branch lengths are drawn proportionally to the amount of changes (scale bar is shown)

To further characterize the new *P. (Haemamoeba)* lineage AENOT11, a phylogenetic tree was constructed with known reference sequences (Fig. 2). As expected, the mosquito isolates identified as *Plasmodium* lineages LINN1, SYAT05, and GRW6 all clustered with high sequence homology (100%, 99–78-100%, and 100%, respectively) with their relevant reference sequences. Similar isolates have been identified in various avian species in New Zealand including isolates of *P. matutinum* LINN1 and *P. vaughani* SYAT05 from New Zealand blackbirds (NZTM2 GenBank HQ454002; NZTM2 GenBank HQ453997) and *P. elongatum* GRW6 isolate from a South Island saddleback (SISB GenBank GU552449)(Fig. 2). The new *P. relictum* isolate AENOT11 clustered with reference sequences generally considered part of the *P. relictum* GRW4 (GenBank AY099041) cluster rather than the closely related *P. relictum* SGS1 (GenBank MZ465355) or New Zealand native isolate KOKAKO01 (GenBank JQ905573). The *P. relictum* GRW4 group includes isolates from around the world, including Madagascar (GenBank MF442547), Japan (GenBank LC230050), USA (GenBank KX867058), South Africa (GenBank KU375974), PADOM10 (MalAvi database), and a New Zealand isolate HIHI01 (GenBank HQ453996). Sequence homology between the new isolate *P. (Haemamoeba)* lineage AENOT11 and the *P. relictum* cluster varied slightly between 99.3 and 99.8%, with the *P. relictum* isolates KOKAKO01 and SGS1 sharing 98.4% and 97.7% homology, respectively, with *P. relictum* AENOT11.

## Discussion

This study is the first detection of avian *Plasmodium* DNA from mosquito thoraxes in New Zealand. *Plasmodium* lineages LINN1, SYAT05, and GRW6 were found in both the abdomen and thorax of the introduced *Ae. notoscriptus* mosquito and the native *Cx. pervigilans mosquito,* suggesting that both are competent vectors for *Plasmodium* spp. *Aedes notoscriptus* (with by far the largest sample collected) was also found to carry a novel *P. (Haemamoeba)* lineage AENOT11 in the abdomen. This study also confirmed the findings made by Gudex-Cross et al. [20] that native mosquitoes outnumber introduced mosquitoes at uninhabited conservation sites, while the introduced species *Cx. quinquefasciatus* and *Ae. notoscriptus* are known to prefer habitats modified by humans and are therefore often found in urban and semi-urban areas [18, 38, 39]. As observed here, *Ae. notoscriptus* was also the most abundant introduced species recorded by Gudex-Cross et al. [20] during a study in the northern North Island of New Zealand.

Sampling methods used to capture mosquitoes are known to influence the species and individual numbers caught. Gudex-Cross et al. [20] emphasized the placement of traps both on the ground and canopy to discern vertical distribution patterns of each species that may be related to feeding patterns and host preference. Different kinds of traps also influence the outcome of a study. Carlson et al. [40] even suggest that conclusions made on the role of vectors by examinations using only a single trapping method should be viewed with caution. While CO_2_-baited light traps such as the ones used in this study collect host-seeking mosquitoes that may feed on a variety of animals, traps baited with readily available birds such as chickens or canaries will accomplish a more specific collection [41]. The possibility of trap bias should be addressed in future studies by using a variety of trapping techniques to consider the specific biology of different New Zealand mosquito species.

During the summer 2012/2013, dry weather with drought conditions in large parts of the North Island negatively impacted the number of mosquitoes caught, particularly at Tiritiri Matangi Island and Mokoia Island in late summer (February 2013), where no mosquitoes were caught. Other similar studies have caught between 40 and 100 individuals per sampling period per site in favorable climatic conditions [23, 42, 43]. Unfortunately, very few mosquitoes were collected on the offshore islands in this study (Hen Island, Cuvier Island, and Tiritiri Matangi Island) during the sampling period. These sites require permits, financial effort, extended logistics, and trips that must be arranged months in advance. As a result, a second trip was not feasible to further examine vector populations on these islands.

To conserve resources and limit expense, mosquitoes of each species were pooled for analysis. As each pool of thorax or abdomen could contain between 1 and 20 individuals, a direct parasite prevalence in each species could not be determined. Therefore, a MIR was calculated for each mosquito species with a PCR-positive thorax and abdomen pool and for a positive thorax pool only. When 23% of total thorax pools is considered, the MIR ranged from 0% to 4.90% depending on the mosquito species examined. Previous studies have shown that the parasite prevalence in thorax samples for other members of the *Culex* species of mosquitoes is variable, and can range widely within species depending on season, year, and location. For example, two studies in Switzerland examining the prevalence in individual thorax samples of *Culex pipiens* found an overall *Plasmodium* spp. prevalence of 6.6% (*n* = 394) in 2006/2007 [44] and 16.3% in 2011, but noted prevalence varied from 1.5% to 20.3% depending on the month sampled [23]. This temporal variation was also noted in similar studies in the USA, Europe, and Japan [43, 45, 46] and should be further investigated in the New Zealand context.

One limitation with only studying vector thoraxes with molecular methods is the possibility that sporozoites, the parasite life stage before transmission to the vertebrate host, can occur in the hemocele during their travel from the midgut to the salivary glands [47]. If the vector is not fully competent, sporozoites may never reach and fully develop in the salivary glands. Consequently, the amplified parasite DNA from mosquito thoraxes may come from noninfectious parasite stages, and therefore non-competent mosquitoes. In addition, to be able to fully confirm the competent vector status of different mosquito species in New Zealand, future studies will have to involve microscopic detection of oocytes in the midgut and sporozoites in the salivary glands of the mosquitoes, followed by experimental transmission studies [44, 48].

With this limitation considered, the results of this study show that the two models including site and mosquito species were significant predictors of *Plasmodium* overall MIR for the two most captured mosquito species sampled, indicating that conditions associated with location and mosquito species are as important in New Zealand as they are in other countries [49–52]. The results for overall and thorax MIR suggest that *Cx. pervigilans*, our native mosquito, with higher MIR, is more likely to transmit avian malaria than *Ae. notoscriptus*. *Culex pervigilans* has long been suspected to be a competent vector for avian malaria in New Zealand [17, 53] and mosquitoes of the genus *Culex* are the most common vectors for these parasites worldwide [5, 41, 44]. However, a similar vector role of other mosquito species in New Zealand cannot be ruled out.

While not all avian *Plasmodium* lineages have a putative vector, members of the *Culex pipiens* complex, which includes the notorious and established vector *Cx. quinquefasciatus* [5], are often featured. For example, the three most common *Plasmodium* lineages found in this study have also been reported in *Cx. pipiens* in several studies on mosquito vectors throughout Europe [23, 44, 45, 54] and in *Cx. pipiens* and *Cx. theileri* in Portugal [55]. To date, mosquito studies in the Pacific region have been limited to Japan, however, the results are consistent with those in Europe with *Plasmodium* lineage SGS1 and PADOM02 detected in the thorax of *Cx. pipiens* and the abdomens of *Aedes albopictus* and *Tripterodie bambusa* [56].

The *Plasmodium* lineages found in mosquitoes during this study are *Plasmodium* (*Huffia*) *elongatum* lineage GRW06, *Plasmodium *sp. LINN1, *P. (Novyella) spp.* lineage SYAT05 and a novel isolate of a *P. (Haemamoeba)* lineage, AENOT11. This is consistent with the most common *Plasmodium* lineages that have been found in New Zealand raptors (*Falco* sp., *Circus* sp., and *Ninox* sp.), little penguins (*Eudyptula minor*), kiwi (*Apteryx* sp.), and a variety of Passeriformes [6, 7, 57, 58]. Both *A. notoscriptus* and *C. pervigilans* carried the *Plasmodium* lineages LINN1, SYAT05, and GRW6 in both the abdomen and the thorax and may be competent vectors for these lineages.

Compared with the number of lineages identified in avian species in New Zealand, diversity appeared to be low during this examination of mosquito vectors. The *Plasmodium* lineages LINN1, SYAT05, and GRW6 were widespread and found on all positive sites around the North Island, except for Cape Kidnappers (only SYAT05 and GRW6) and Hen Island (only SYAT05). The *Plasmodium (Haemamoeba)* lineage AENOT11 was only identified in Palmerston North, which was most likely due to low sample sizes at other locations, especially from the island sites. In contrast, an examination of *Plasmodium* spp. diversity among introduced birds with overlapped ranges with the threatened North Island Saddleback (*Philesturnus carunculatus rufusater*) revealed six distinct avian *Plasmodium* lineages, including the three cosmopolitan lineages SYAT05, LINN1, and GRW6 at many of the same locations as the present study [59].

In addition, the low diversity of collected lineages might also be connected to sampling only being performed during late summer and early autumn. Lalubin et al. [23] have reported seasonal changes in lineage composition throughout the year, where SYAT05 decreased from spring to summer in favor of three lineages of the *P. relictum* group (SGS1, GRW11, and PADOM02). Similar seasonal changes have also been observed by Kim and Tsuda [60] in Japan, where they found a temporal association with a high prevalence of *Plasmodium* lineages infecting the two dominant mosquito species and the transmission season. In future studies in New Zealand, a wider range of seasons should be considered to mitigate possible seasonal variations in *Plasmodium* lineage composition.

## Conclusions

This study identified four *Plasmodium* lineages, LINN1, SYAT05, GRW6, and a new *P. (Haemamoeba)* lineage AENOT11 in the mosquitoes tested, with the first three in both abdomens and thoraxes of introduced *A. notoscriptus* and native *C. pervigilans*. These mosquitoes are therefore likely competent vectors for avian malaria in New Zealand and found in high abundance at all sampled sites. With site and mosquito species being significant predictors of *Plasmodium* overall MIR, our findings support the hypothesis that at least one native and one introduced mosquito species are competent vectors for introduced *Plasmodium* lineages in New Zealand. This study provides the first step to improving our understanding of mosquito transmission of *Plasmodium* species in New Zealand and will lead to improving our understanding of risk for native avifauna.

## Data Availability

The *Plasmodium* species *cytochrome b* gene sequences were submitted to the GenBank database under the reference nos. OR050956-OR050966.

## Abbreviations

MIR: Minimum infection rate
AIC: Akaike information criterion
GLM: Generalized linear models
PCR: Polymerase chain reaction
qPCR: Real-time PCR
